# Supplementary material for: COSUTI: A Core Outcome Set (COS) for Interventions for the Treatment of Uncomplicated Urinary Tract Infection (UTI) in Adults
Source: Antibiotics (Basel). 2022 Dec 19;11(12):1846. doi: 10.3390/antibiotics11121846 (PMC9774367; doi:10.3390/antibiotics11121846)
Supplement: Supplementary file 1 [file antibiotics-11-01846-s001.zip › antibiotics-2056130-supplementary.pdf]

COSUTI

CORE OUTCOME SET FOR THE TREATMENT OF URINARY TRACT INFECTIONS

Supplementary material S1

Outcomes included in round 1 Delphi survey

No.	Domain	Outcome	Plain English definition
1	***Treatment***	The use of any antibiotic up to day 30	The use of any antibiotics by a patient up to day 30
2		Use of antibiotics	The use of any antibiotics by a patient at any time
3		Duration of treatment	The length of time a patient's treatment (e.g. taking antibiotics) lasts
4	***Clinical Cure***	Clinical cure	No signs or symptoms of UTI
5		Clinical cure by day 3 from initiation of treatment	No signs or symptoms of UTI by day 3 from when treatment was started
6		Clinical cure by day 7 from initiation of treatment	No signs or symptoms of UTI by day 7 from when treatment was started
7		Clinical cure by day 14 from initiation of treatment	No signs or symptoms of UTI by day 14 from when treatment was started
8	***Symptom Resolution***	Number of days to symptom resolution	The number of days it takes before a patient is no longer experiencing symptoms of a UTI. Symptoms are experienced or felt by a patient themselves and suggest that they might have a UTI, for example a strong, persistent urge to pass urine or burning sensation when passing urine
9		Resolution of clinical signs or symptoms	A patient is no longer showing clinical signs, or experiencing symptoms of a UTI
10		Symptom resolution on Day 3	A patient is no longer experiencing symptoms on day 3
11		Symptom resolution on Day 7	A patient is no longer experiencing symptoms on day 7
12		Short-term symptomatic cure: the absence of urinary symptoms up to two weeks after start of treatment.	A patient is no longer experiencing symptoms of a UTI at any time up to two weeks after treatment has started
13		Long-term symptomatic cure: the absence of urinary symptoms up to eight weeks after start of treatment.	A patient is no longer experiencing symptoms of a UTI at any time up to eight weeks after treatment has started
14		Relative improvement in clinical UTIs signs and symptoms	A patient's signs and symptoms have slightly improved to some extent

15		Persistence or progression of any clinical UTIs signs or symptoms or appearance of new signs or symptoms	UTI clinical signs or symptoms stay the same or get worse for a patient, or new clinical signs or symptoms of a UTI appear
16		Burden of symptoms	How much of a problem symptoms are for a patient
17		Burden of symptoms on Day 3	How much of a problem symptoms are for a patient on day 3
18		Burden of symptoms on Day 7	How much of a problem symptoms are for a patient on day 7
19		Absence of pyuria	The absence of pus in the urine
20	***Symptoms***	Duration of symptoms	The length of time symptoms last
21		Duration of moderately bad symptoms	The length of time that symptoms, that are moderately bad, last
22		Duration of symptoms until little or no problem	The length of time that symptoms, that are little to no problem, last
23		Symptom severity	How serious/ uncomfortable symptoms are
24		Symptom severity on days 2–4	The seriousness of symptoms on days 2- 4
25		Changes in clinical symptoms	A change in the symptoms experienced by a patient
26		Early symptoms by days 1 to 4	Symptoms that occur early in the lifetime of a UTI (by days 1 to 4)
27		Late symptoms by days 5 to 10	Symptoms that occur later in the lifetime of a UTI (by days 5 to 10)
28		Symptom scores, (none, mild moderate or severe)	The score given by a patient to rate how serious a symptom is for them
29		Total symptom burden	How much discomfort the symptoms have caused a patient
30	***Complications***	Febrile UTI	A UTI with the additional symptom of a high body temperature (temperature due to the UTI)
31		Pyelonephritis	One or both the kidneys become infected. The infection can be caused by bacteria or a virus
32		Development of pyelonephritis during treatment	One or both the kidneys become infected while a patient is being treated for their UTI
33		Development of pyelonephritis after treatment	One or both the kidneys become infected after a patient has completed treatment for their UTI
34		Pyelonephritis within 28 days	One or both the kidneys become infected within 28 days
35		Episodes of suspected upper UTI	The number of times a patient is suspected to be experiencing a UTI in the upper urinary tract (i.e., the kidneys and a tube called the ureter which connects the kidneys to the bladder)
36		Complicated UTI	A complicated UTI is a more severe form of a UTI that has possibly extended beyond just the involvement of the bladder

37		Worsening of UTI, progression to complicated UTI	The signs and/or symptoms of a patients UTI gets worse and leads to a complicated UTI
38		Urosepsis	Where the bacteria that caused the UTI infects a person's bloodstream
39		Urosepsis within 28 days	Urosepsis that develops within 28 days
40		Other renal complications	Any other complications of the urinary system that may arise from a patient having a UTI
41		Septic syndrome	An infection associated with a number of other indicators or symptoms suggesting a severe infection
42		Septicaemia	Septicaemia is the clinical name for bacterial infection of the blood stream
43		Hospitalisation/hospital admissions	Admission of a patient to hospital for treatment of a UTI or complications of a UTI
44		Need for intravenous antibiotics	The need for a patient to receive antibiotics that are given directly into a vein so that they can enter the bloodstream immediately and bypass the absorption in the gut
45		Life threatening or associated with permanent disability	An illness that is so serious that it threatens the life of a patient, or that has the potential to cause permanent disability
46		Death	End of life
47	***Adverse treatment reactions***	Gastrointestinal symptoms (e.g. nausea)	Symptoms, such as nausea, that are experienced by patients in the gastrointestinal tract, from the mouth to the rectum
48		Gastrointestinal haemorrhage	Bleeding anywhere in tract from the mouth to the rectum
49		Vomiting	Uncontrolled emptying of the stomach through the mouth
50		Abdominal pain	Pain experienced by a patient in the abdominal area (below the ribs and above the pelvis)
51		Diarrhoea	Unwanted and frequent passing of watery or loose stool
52		Skin rash	Skin that becomes scaly, bumpy, itchy, or otherwise irritated
53		Skin findings (including severe reactions such as Stevens–Johnson syndrome)	A noticeable irregularity on a patients skin
54		Exfoliative dermatitis	Widespread redness of the skin and scaling of the skin
55		Facial skin alteration	A change to the skin on a patient's face
56		Vaginal discomfort	Itching or irritation in the vagina
57		Vaginitis	Vaginitis is a word for various disorders that cause inflammation or infection of the vagina.
58		Vaginal candidiasis	A vaginal yeast infection (vaginal candidiasis) is a fungal infection that causes irritation, discharge and intense itchiness of the vagina and the vulva
59		Neurological events	Events or instances that involve the central and peripheral nervous system
60		Headache	

61		Light-headedness	A feeling of dizziness or as if you might faint
62		Disturbance in attention	Difficulties with attention span
63		Restlessness	Being unable to remain still
64		Haematological event	Events or instances that involve the blood
65		Pulmonary and hepatic fibrosis	Damage and scarring to the tissue of the lung and liver
66		Epigastralgy	Pain in the upper central region of the abdomen for example, heartburn or reflux
67		Nephrotoxicity	Toxicity (the state of being poisonous or damaging) in the kidneys
68		Ototoxicity	Toxicity in the ear
69		Dyspepsia	Indigestion
70		Decreased appetite	No interest in food
71	***Relapse***	Frequency of relapses until day 28 after initial resolution of symptoms	How often symptoms come back or get worse in the 28 days after original symptoms had improved.
72		Relapse after initial resolution of symptoms	When symptoms come back or get worse after they had originally improved.
73		Early relapse of symptoms by day 14 after initial resolution of symptoms	When symptoms come back or get worse within 14 days of symptoms originally improving.
74		Frequency of relapse or new infection by day 15 after initial resolution of symptoms	How often symptoms come back, get worse or a new infection begins within 15 days of symptoms originally improving.
75		Frequency of relapse or new infection by day 28 after initial resolution of symptoms	How often symptoms come back, get worse or a new infection begins within 28 days of symptoms originally improving.
76		Incidence of new symptoms of UTI after initial clinical cure	How often new symptoms of an infection of the urinary bladder occurs after it had originally improved
77		Recurrent UTI after initial resolution of symptoms up to day 28	Symptoms come back or get worse within 28 days of originally improving.
78		Recurrent UTI after initial resolution of symptoms within six and 12 months	Symptoms come back or get worse within 6 to 12 months of originally improving.
79		Recurrent UTI after initial resolution of symptoms 12 months	Symptoms come back or get worse within 12 months of originally improving.
80		Recurrence 4– 6 weeks following treatment completion	Signs or symptoms come back or get worse 4- 6 weeks after treatment is completed
81		Clinical Recurrence of signs after initial resolution of symptoms	Signs come back or get worse after originally improving
82		Recurrence of a UTI within 6 months after initial resolution of symptoms	Signs or symptoms come back or get worse within 6 months after originally improving
83		Clinical recurrence within 30 days	The frequency of which clinical signs of a UTI come back or get worse within 30 days

84		Clinical recurrence within 90 days	The frequency of which of clinical signs of a UTI come back or get worse within 90 days
85	***Failure, discontinuation and tolerance***	The persistence of signs and symptoms at the post treatment visit	Signs and symptoms stay or get worse following the completion of treatment
86		The persistence of signs and symptoms at the post treatment visit on the first day following completion of treatment	Signs and symptoms have remained or gotten worse since completion of treatment from the first visit to the medical practitioner
87		The persistence of signs and symptoms at the post treatment visit at 7 to 10 days	Signs and symptoms have remained or gotten worse at the visit to the medical practitioner at 7 to 10 days following completion of treatment
88		The persistence of signs and symptoms at the post treatment visit at 8 weeks	Signs and symptoms have remained or gotten worse at the visit to the medical practitioner at 8 weeks following completion of treatment
89		Number of return visits to the GP with UTI	The number of times a patient returns to their General Practitioner (GP) for a visit due to UTI
90		Re-consultation in 1 month with UTI	Repeat appointment with medical practitioner within 1 month due to UTI
91		Re-consultation in 3 months with UTI	Repeat appointments in 3 months due to UTI
92		Secondary antibiotic treatments	The use of a second antibiotic to treat the UTI following failure of the first antibiotic to successfully treat it
93		Discontinuation of treatment	The decision not to continue with treatment by a medical practitioner
94		Treatment adherence	Whether a patient complies with taking the medications that have been prescribed to them by a medical practitioner to treat a UTI
95	***Quality of life & Patient Satisfaction***	Clinical reported quality of life	A patient's quality of life, as reported by a trained healthcare practitioner after they observe a patient’s health condition
96		Patient self-reported quality of life	A patient's quality of life, as reported by the patient themselves
97		Days absent from work	The number of days that the patient was unable to attend work due to UTI signs or symptoms
98		Functional status	An individual's ability to perform normal daily activities required to meet basic needs, fulfil usual roles, and maintain health and well-being
99		Long-term mortality, caused by and related to UTI	Death occurring a long period of time after but due to a patient's UTI
100		Mental health status	The condition of a patient's mental health
101		Activity impairment on days 1-7	Reduction in a patient's activity in the first week
102		Overall satisfaction with management of the UTI	A patient's level of contentment or happiness with the way healthcare staff have organised the care of their UTI

103		Patients' satisfaction with the treatment of the UTI	A patient's level of contentment or happiness with the treatment of their UTI by their healthcare practitioners
104	***Antibiotic Resistance, Bacterial Cure & Microbiological Cure***	Development of antibiotic resistance	Antibiotic resistance occurs when bacteria change in some way that reduces or stops how well drugs, chemicals, or other agents, that are designed to cure or prevent UTIs, work
105		Short-term bacteriological cure within 14 days of the start of treatment	The results of testing of a patient's urine sample at their first follow-up appointment with a medical practitioner within 14 days of the start of treatment suggests that the UTI has been cured
106		Long-term bacteriological cure within 30 days of the start of treatment	The results of testing of a patient's urine sample at their first follow-up appointment with a medical practitioner within 30 days of the start of treatment suggests that the UTI has been cured
107		Bacteraemia up to one year	Bacteria is found in a patient's blood up to one year after initial presentation for UTI treatment
108		The microbiological cure rates at 5-9 days after treatment	The number of patients whose urine tests negative for the bacteria related to UTIs 5-9 days after treatment
109	***Microbiological Relapse & Microbiological Failure***	microbiological recurrence within 30 days	The number of patients whose urine tests positive for the bacteria related to UTIs within 30 days of testing negatively
110		microbiological recurrence within 90 days	The number of patients whose urine tests positive for the bacteria related to UTIs within 90 days of testing negatively
111		Bacteriological/microbiological relapse	A patient who initially tested negative for the bacteria related to UTIs then experienced a growth by day-28 of that same bacteria
112		The persistence of causative organism in urine post treatment	The cause of the UTI remains in a patients' urine when tested after treatment
113		The persistence of causative organism in urine at the post treatment visit on the first day following completion of treatment	The cause of the UTI remains in a patients' urine when tested at the visit to the medical practitioner on the first day following completion of treatment
114		The persistence of causative organism in urine at the post treatment visit at 7 to 10 days	The cause of the UTI remains in a patients' urine when tested at the visit to the medical practitioner at 7- 10 days following completion of treatment
115		The persistence of causative organism in urine at the post treatment visit at 8 weeks	The cause of the UTI remains in a patients' urine when tested at the visit to the medical practitioner at 8 weeks following completion of treatment
116		The microbiological outcome 4–6 weeks after treatment completion demonstrated UTI recurrence.	The reoccurrence of a bladder infection 4-6 weeks after treatment was completed
